# Effect of prior receipt of antibiotics on the pathogen distribution and antibiotic resistance profile of key Gram-negative pathogens among patients with hospital-onset urinary tract infections

**DOI:** 10.1186/s12879-017-2270-7

**Published:** 2017-02-28

**Authors:** Monique R. Bidell, Melissa Palchak Opraseuth, Min Yoon, John Mohr, Thomas P. Lodise

**Affiliations:** 1 Albany College of Pharmacy and Health Sciences, 106 New Scotland Avenue, Albany, 12208-3492 NY USA; 2 Merck & Co., Inc., Kenilworth, NJ USA

**Keywords:** Antibiotic use, Antimicrobial resistance, Fluoroquinolones, Gram-negative pathogens, Multidrug resistant, Urinary tract infection

## Abstract

**Background:**

This retrospective cohort study characterized the impact of prior antibiotic exposure on distribution and nonsusceptibility profiles of Gram-negative pathogens causing hospital-onset urinary tract infections (UTI).

**Methods:**

Hospital patients with positive urine culture for *Pseudomonas aeruginosa, Escherichia coli, Klebsiella pneumoniae,* and other Enterobacteriaceae ≥3 days after hospital admission were included. Assessment outcomes included the distribution of bacteria in urine cultures, antibiotic susceptibility patterns, and the effect of prior antibiotic exposure, defined as 0, 1, or ≥2 prior antibiotics, on the distribution and antibiotic susceptibility profiles of the Gram-negative organisms.

**Results:**

The most commonly isolated pathogens from 5574 unique UTI episodes (2027 with and 3547 without prior antibiotic exposure) were *E. coli* (49.5%), *K. pneumoniae* (17.1%), and *P. aeruginosa* (8.2%). *P. aeruginosa* was significantly more commonly isolated in patients with ≥2 prior antibiotic exposures (12.6%) compared with no exposure (8.2%; *p* = 0.036) or 1 prior exposure (7.9%; *p* = 0.025). Two or more prior antibiotic exposures were associated with slightly higher incidences of fluoroquinolone nonsusceptibility, multidrug resistance, and extended-spectrum β-lactamase phenotype compared with 0 or 1 exposure, suggesting an increased risk for resistant Gram-negative pathogens among hospital patients with urinary tract infections occurring ≥3 days after admission.

**Conclusions:**

Clinicians should critically assess prior antibiotic exposure when selecting empirical therapy for patients with hospital-onset urinary tract infections caused by Gram-negative pathogens.

**Electronic supplementary material:**

The online version of this article (doi:10.1186/s12879-017-2270-7) contains supplementary material, which is available to authorized users.

## Background

Increasing antibiotic resistance among Gram-negative pathogens, particularly in the hospital setting, is well documented and constitutes a major public health concern [1, 2]. Gram-negative organisms are implicated in a number of hospital-acquired infections, with urinary tract infections (UTIs) particularly common [1, 3]. Antibiotic resistance among hospital-acquired infections owing to Gram-negative pathogens greatly complicates the administration of timely and appropriate therapy, placing patients at increased risk for deleterious outcomes [4–6]. Resources such as antibiograms can facilitate the empirical antimicrobial selection process [7] by characterizing local resistance patterns. However, reliance on a single collective isolate institutional antibiogram for empirical antibiotic selection is associated with several notable shortcomings. Collective isolate antibiograms developed by many institutions do not capture the distribution of pathogens associated with a particular infection, nor do they reflect the susceptibility profile of a particular pathogen at an infection site (eg, urinary tract). Although culture site–specific antibiograms can ameliorate this issue, they do not indicate patient-specific factors that increase the likelihood of an antibiotic-resistant infection [7].

Two critical patient characteristics that modify the risk for antibiotic-resistant infection are recent antibiotic exposure and recent admission to, or current residence in, a health care institution [2, 8, 9]. Considering the limitations associated with an antibiogram-only approach to empirical antibiotic selection, this study was designed to characterize the impact of prior antibiotic exposure on the distribution and nonsusceptibility profiles of key Gram-negative pathogens among US inpatients with hospital-onset UTIs. We specifically focused on this single modifiable risk factor because it is readily identifiable and accessible in the medical record system for all patients. Other possible patient risk factors were not included in the analysis so that a simple and straightforward guide to empirical antibiotic selection could be created.

## Methods

### Study design and population

This retrospective, observational study used hospital discharge data from the Premier Healthcare Database, which at the time of this study contained data from more than 435 million patient encounters. Laboratory results were available from a subset of approximately 160 facilities in the Premier Healthcare Database. Patients were included in the study if all the following criteria were met: inpatient discharge between January 1, 2012, and March 31, 2013; positive urine culture for any of the prespecified Gram-negative bacteria ≥3 days after hospital admission; and receipt of an antibiotic with activity against Gram-negative pathogens on the index culture date or within the 3-day period thereafter. The first documented urine culture was included in the analysis. Duplicate isolates from subsequent urine cultures within 30 days were excluded. Duplicate isolates recovered from urine cultures >30 days after the index culture were included because we felt these to be representative of either recurrent or new infection. As such, we felt it was important to include these subsequent occurrences as unique episodes within the study.

The Gram-negative organisms of interest were *Escherichia coli*, *Klebsiella pneumoniae*, *Pseudomonas aeruginosa,* and other members of the Enterobacteriaceae family (excluding *E. coli* and *K. pneumoniae*)*.* Prior antibiotic exposure was defined as administration of ≥1 prespecified agents with Gram-negative activity during the current hospitalization and before the index urine culture. Antibiotics included for assessment of prior exposure were those available in the working Premier data set: meropenem, doripenem, imipenem, ertapenem, piperacillin/tazobactam, cefepime, ceftazidime, ceftriaxone, cefotaxime, ciprofloxacin, levofloxacin, gentamicin, tobramycin, amikacin, ampicillin/sulbactam, cefazolin, gatifloxacin, cefazolin, tigecycline, and ticarcillin/clavulanic acid (or clavulanate). Data for other groups of antibiotics, including second-generation cephalosporins and trimethoprim/sulfamethoxazole, were not available.

### Outcomes

The first outcome measure was distribution and antibiotic susceptibility patterns among the prespecified Gram-negative organisms for patients meeting inclusion criteria. The second outcome measure was the association of prior antibiotic exposure, defined as 0, 1, or ≥2 prior exposures, with the distribution and antibiotic susceptibility profiles of the Gram-negative organisms of interest. Organisms were assessed for susceptibility to fluoroquinolones (ciprofloxacin, levofloxacin, gatifloxacin), carbapenems (meropenem, imipenem, doripenem, ertapenem), and piperacillin/tazobactam.

For the purposes of this analysis, antibiotic susceptibility was classified as susceptible versus nonsusceptible (ie, intermediate or resistant), based on the microbiology report. Multidrug resistance was defined as nonsusceptibility to ≥1 agents in ≥3 antibiotic classes [10]. For *P. aeruginosa*, only meropenem-, imipenem-, or doripenem-nonsusceptibility was used to define carbapenem resistance; *P. aeruginosa* isolates were considered nonsusceptible to third-generation cephalosporins if they were resistant to ceftazidime, or, if data were unavailable, to cefepime. For Enterobacteriaceae, isolates were considered nonsusceptible to a third-generation cephalosporin if they had documented resistance to at least two agents (ceftriaxone, ceftazidime, or cefotaxime). Nonsusceptibility to third-generation cephalosporins was considered indicative of an extended-spectrum beta-lacatmase (ESBL) phenotype. For patients with missing carbapenem, piperacillin/tazobactam, or MDR susceptibility data, isolates were considered carbapenem-susceptible, piperacillin/tazobactam-susceptible, or non-MDR if they were susceptible to a third-generation cephalosporin. Otherwise, carbapenem, piperacillin/tazobactam, and MDR susceptibility data were considered missing and were not included in the analyses that evaluated the relationship between prior antibiotic exposures and antibiotic susceptibility profiles.

### Statistical analysis

Unadjusted descriptive analysis was used to characterize the distribution and antibiotic susceptibility patterns of the Gram-negative organisms of interest and the effect of prior antibiotic exposure on each of these patterns. Although the study was not powered for prespecified statistical analyses, possible significance between groups (≥2 prior exposures compared with 0 or 1 prior exposure) was calculated using the method of Miettinen and Nurminen.

## Results

### Overall pathogen distribution and resistance profile

**Fig. 1:**
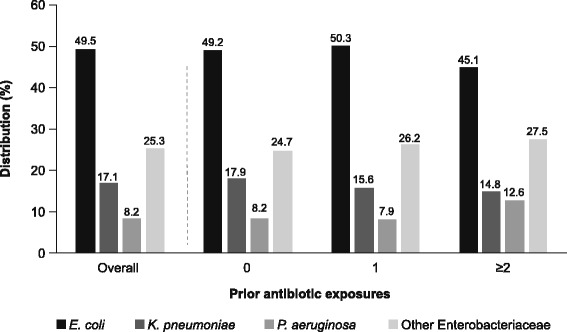
Pathogen distribution by prior antibiotic exposure

**Fig. 2:**
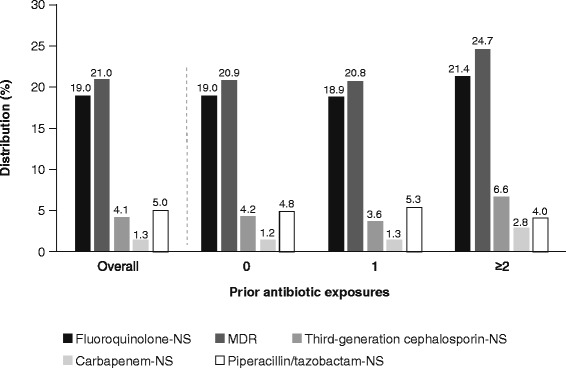
Antibiotic nonsusceptibility phenotypes across all pathogens by prior antibiotic exposure. *MDR* multidrug resistant; *NS* nonsusceptible

Descriptive statistics of patient demographics and characteristics of hospital visits are provided (see Additional file 1). A total of 5574 unique UTI episodes were included in the analysis (*n* = 2027 prior antibiotic exposure; *n* = 3547 no prior exposure), from which 6093 pathogens were isolated (*n* = 2227 from patients with prior antibiotic exposure; *n* = 3866 from patients with no prior exposure). The most commonly isolated pathogens were *E. coli* (*n* = 3013; 49.5%), *K. pneumoniae* (*n* = 1039; 17.1%), and *P. aeruginosa* (*n* = 502; 8.2%) (Fig. 1). Among all pathogens, fluoroquinolone nonsusceptibility and multidrug resistance exceeded 19 and 21%, respectively (Fig. 2). The ESBL phenotype was noted in 4.1% of pathogens, and carbapenem nonsusceptibility was noted in <2%.

### Effect of prior antibiotic exposure

The distribution of pathogens in relation to prior antibiotic exposure is shown in Fig. 1. *P. aeruginosa* was isolated significantly more often in patients with ≥2 prior antibiotic exposures (12.6%) than in patients with no prior antibiotic exposure (8.2%; 95% confidence intervals [CI] 0.2, 10.1; *p* = 0.036) or with 1 prior exposure (7.9%; 95% CI 0.5, 10.5; *p* = 0.025). For other pathogen groups, the presence or absence of prior antibiotic exposure did not substantially affect distribution trends, and differences were nonsignificant.

Nonsusceptibility characteristics with respect to 0, 1, or ≥2 prior antibiotic exposures are shown in Fig. 2 and Table 1. Among all pathogens, a trend toward slightly higher incidences of fluoroquinolone nonsusceptibility and of multidrug-resistant and ESBL phenotypes wase seen with ≥2 prior antibiotic exposures than with 1 or 0 (Fig. 2); however, with the exception of ESBL phenotype in patients with ≥2 vs 1 prior antibiotic exposures (6.6% vs 3.6%; 95% CI 0.1, 7.7; *p* = 0.041), these differences in incidence were not statistically significant. This trend seemed to be driven largely by susceptibility pattern changes observed with *K. pneumoniae* (Table 1).

**Table 1:** Nonsusceptibility phenotypes by prior antibiotic exposure

Pathogen	Nonsusceptibility phenotype	Overall, % NS (*n*/*N*)	Prior antibiotic exposure		
			0	1	≥2
*Escherichia coli* (*n* = 3013)^a,b^	Fluoroquinolone-NS	25.4 (758/2990)	25.5 (482/1888)	24.8 (253/1020)	28.0 (23/82)
	Third-generation cephalosporin-NS	3.5 (105/3013)	3.8 (72/1903)	2.7 (28/1028)	6.1 (5/82)
	MDR	23.4 (705/3013)	24.1 (458/1903)	21.6 (222/1028)	30.5 (25/82)
	Carbapenem-NS	0.1 (4/3006)	0.2 (3/1900)	0.1 (1/1025)	0 (0/81)
	Piperacillin/tazobactam-NS	4.4 (131/2965)	4.6 (86/1871)	4.1 (42/1014)	3.8 (3/80)
*Klebsiella pneumoniae* (*n* = 1039)^a,c^	Fluoroquinolone-NS	6.8 (70/1036)	6.0 (41/689)	7.5 (24/320)	18.5 (5/27)
	Third-generation cephalosporin-NS	3.7 (38/1039)	2.7 (19/692)	5.0 (16/320)	11.1 (3/27)
	MDR	10.1 (105/1039)	9.2 (64/692)	11.3 (36/320)	18.5(5/27)
	Carbapenem-NS	1.5 (16/1035)	1.0 (7/690)	2.5 (8/319)	3.8 (1/26)
	Piperacillin/tazobactam-NS	7.1 (73/1021)	6.0 (41/685)	9.4 (29/310)	11.5 (3/26)
*Pseudomonas aeruginosa* (*n* = 502)^a,d^	Fluoroquinolone-NS	21.8 (109/499)	22.2 (70/316)	20.6 (33/160)	26.1 (6/23)
	Third-generation cephalosporin-NS	8.8 (44/502)	10.4 (33/318)	5.0 (8/161)	13.0 (3/23)
	MDR	8.8 (44/502)	10.4 (33/318)	5.0 (8/161)	13.0 (3/23)
	Carbapenem-NS	7.3 (36/496)	7.3 (23/314)	5.7 (9/159)	17.4 (4/23)
	Piperacillin/tazobactam-NS	4.0 (20/494)	4.1 (13/314)	4.4 (7/158)	0 (0/22)
Other Enterobacteriaceae (*n* = 1539)^a,e^	Fluoroquinolone-NS	14.1 (216/1531)	14.5 (137/947)	13.9 (74/534)	10.0 (5/50)
	Third-generation cephalosporin-NS	3.9 (60/1539)	4.0 (38/953)	3.9 (21/536)	2.0 (1/50)
	MDR	27.6 (424/1539)	26.4 (252/953)	29.9 (160/536)	24.0 (12/50)
	Carbapenem-NS	1.5 (23/1525)	1.5 (14/944)	1.7 (9/532)	0 (0/49)
	Piperacillin/tazobactam-NS	4.9 (75/1529)	4.8 (45/947)	5.4 (29/533)	2.0 (1/49)

*MDR* multidrug resistant, *n* number of nonsusceptible isolates, *N* total number of isolates, *NS* nonsusceptible

^a^For patients with missing susceptibility data, isolates were considered to be carbapenem-susceptible, piperacillin/tazobactam-susceptible, or non-MDR if they were susceptible to a third-generation cephalosporin. Otherwise, carbapenem, piperacillin/tazobactam, and MDR susceptibility data were considered missing, and isolates were not included in the analyses

^b^ *E. coli*: 903 isolates were missing carbapenem susceptibility data, of which 896 were classified according to the rules described above and seven were excluded from the analysis due to missing data; 1412 isolates were missing piperacillin/tazobactam susceptibility data, of which 1364 were classified according to the rules and 48 were excluded

^c^ *K. pneumoniae*: 317 isolates were missing carbapenem susceptibility data, of which 313 were classified according to the rules and four were excluded; 443 isolates were missing piperacillin/tazobactam susceptibility data, of which 425 were classified according to the rules and 18 were excluded

^d^ *P. aeruginosa*: 97 isolates were missing carbapenem susceptibility data, of which 91 were classified according to the rules and six were excluded; 109 isolates were missing piperacillin/tazobactam susceptibility data, of which 101 were classified according to the rules and eight were excluded

^e^Other Enterobacteriaceae: 494 isolates were missing carbapenem susceptibility data, of which 480 were classified according to the rules and 14 were excluded; 721 isolates were missing piperacillin/tazobactam susceptibility data, of which 711 were classified according to the rules and ten were excluded

## Discussion

The aim of this study was to characterize the distribution and antibiotic nonsusceptibility profiles of key Gram-negative organisms by the presence of antibiotic exposures among patients with hospital-onset UTIs. Although numerous patient factors can affect pathogen distribution and susceptibility trends, we focused on this one risk factor given that it is modifiable and easily evaluated by clinicians using the medical record. Antibiotic exposure is an important consideration given a recent multicenter prevalence study describing antibiotic use in 50% of inpatients on any given day during admission [11]. Therefore, our approach focusing on the number of exposures offers a simple and straightforward supplement to guide empirical antibiotic selection for hospital-onset UTI treatment.

Our study focusing on the single risk factor of antibiotic exposure produced several notable findings. Consistent with epidemiologic trends, *E. coli* (49.5%) was the most commonly isolated pathogen [8, 9, 12], and this was consistent across all categories of prior antibiotic exposure. Although the simple binary designation of prior antibiotic exposure did not seem to affect overall pathogen distribution or nonsusceptibility trends, differences became apparent on stratification by number of exposures. Prior receipt of ≥2 antibiotic regimens was associated with a significantly higher frequency of *P. aeruginosa* UTIs and with slightly higher incidences of fluoroquinolone-, carbapenem-, and third-generation cephalosporin-nonsusceptibility as well as multidrug resistance. Collectively, our findings suggest that previous exposure to ≥2 antibiotic regimens is associated with an alteration of the distribution and susceptibility profiles of Gram-negative pathogens among patients with hospital-onset UTIs.

Although antibiotic nonsusceptibility was highest among those with ≥2 prior exposures to antibiotics, fluoroquinolone nonsusceptibility rates were >18%, even in the absence of prior antibiotic exposure. This finding is not unexpected given the large-scale use of fluoroquinolones for UTIs in both community and health care settings. Emergence of resistance to these antibiotics is recognized by a number of world health agencies and is considered a major public health concern [1, 2, 12]. Because practice guidelines recommend empirical use of fluoroquinolones for UTIs only if local drug resistance rates do not exceed 10% [12], our findings suggest that caution should be exercised with empirical use of fluoroquinolones for hospital-onset UTIs, and strong consideration should be given to alternative therapies with broad-spectrum, Gram-negative coverage empirically.

Our findings also highlight the limitations associated with relying solely on antibiograms for patients with hospital-onset UTI, particularly those with ≥2 antibiotic exposures. Although antibiograms are a useful starting point in the empirical drug selection process, they reflect the cumulative susceptibility rates for the first recovered pathogen in a particular patient, regardless of current site, timing of collection (community vs hospital onset), or patient-specific risk factors for resistance (eg, prior exposure to antibiotics) [7]. Recent guidelines from the Infectious Diseases Society of America and the Society for Hospital Epidemiology of America acknowledge several of these limitations and encourage the development of enhanced antibiograms stratified by various parameters (eg, age, location within the institution, infection site, patient comorbidities, and site of acquisition) to improve empirical selection of antibiotics. Furthermore, these guidelines encourage the development of institution-specific clinical treatment guidelines for common infectious diseases [13]. Our findings that prior antibiotic exposure alters the distribution of pathogens and the susceptibility profiles of common Gram-negative organisms implicated in UTIs occurring ≥3 days after admission can easily be incorporated into clinical treatment guidelines to promote a more patient-specific approach to timely selection of appropriate empirical antibiotic therapy. In addition to guiding the selection of appropriately broad-spectrum empirical therapy for hospital-onset UTI, our findings may be helpful to institutional antimicrobial stewardship programs to target antibiotics likely to be inappropriate (eg, fluoroquinolones).

Some limitations should be considered when interpreting these findings. First, though we acknowledge that several factors may contribute to antibiotic-resistant infections, we purposefully did not assess multiple risk factors because data were not uniformly available for all patients. We also believed that inclusion of other patient factors would complicate our intent to create a simple, straightforward approach to guide empirical antibiotic selection. Because we did not adjust for other factors, our findings cannot substantiate prior use of antibiotics as an independent risk factor for, or as a cause of, resistance, but our findings do show that prior use is an additional easily identifiable variable that can be used to guide empirical therapy. Second, the study was restricted to patients with UTIs occurring ≥3 days after hospital admission. Although we had access to detailed hospitalization data, information on antibiotic and health care exposure before admission was limited. As such, we focused on patients for whom complete, detailed data were available. The 3-day time frame after hospital admission fails to account for recent antibiotic use in outpatient or other institutional settings; therefore, the potential effects of these exposures on our findings are unknown. Third, this study did not specifically quantify the relationship between cumulative duration of exposure to all or any given antibiotic and the presence of resistance. Given that the goal of the study was to provide clinicians with a straightforward, easily adaptable method for empirical antibiotic selection, we did not think it was necessary to specify with increased granularity the relationship between duration of prior antibiotic exposure and resistance. Although we were able to document prior receipt of many commonly used antibiotics with Gram-negative activity, data for other groups of antibiotics, including second-generation cephalosporins, and trimethoprim/sulfamethoxazole, were not available for this analysis. The relatively small number of patients with ≥2 prior antibiotic exposures might also limit the interpretation of findings regarding resistance, though increased rates observed among fewer pathogens is still of concern. In addition, though the statistical analysis suggests some significant differences between patients with ≥2 prior exposures and patients with 0 or 1 prior exposure, it should be noted that the numbers of patients in some of the groups were very small. As such, determination of the true statistical relevance of prior antibiotic exposure will require additional studies adequately powered to determine significance levels. Fourth, given that the presence of the ESBL phenotype among pathogens was not formally tested, the use of select third-generation cephalosporin resistance as a marker for ESBL may underestimate or overestimate the true incidence of ESBL. However, we believe third-generation cephalosporin resistance represented a practical surrogate because it is readily available to clinicians on urine culture susceptibility reports and that routine formal ESBL testing is not recommended by the Clinical and Laboratory Standards Institute (CLSI). Furthermore, given that culture susceptibility reports were from clinical and not reference laboratories, it can be assumed that third-generation cephalosporin resistance was reported using the US Food and Drug Administration–approved minimum inhibitory concentration breakpoint, which may better correlate with the presence of ESBL than CLSI breakpoints [14]. Finally, because findings are derived from positive urine cultures in the absence of a complete clinical picture, it is difficult to definitively distinguish between true infections and colonization. However, all patients received an antibiotic in response to a positive urine culture, which is suggestive of symptomatic infection for most patients.

## Conclusions

Having received ≥2 prior antibiotic regimens was associated with increased risk for a resistant Gram-negative pathogen among patients with hospital-onset UTIs. In addition to considering the hospital antibiogram, which should be stratified for greater usefulness, hospital clinicians should critically assess prior exposure to antibiotics when selecting empirical therapy for patients with UTIs that occur ≥3 days after admission. Awareness of common pathogen distribution and nonsusceptibility trends among hospital patients with UTIs, combined with readily identifiable patient risk factors for infection with resistant pathogens, can provide a useful framework for clinicians during the empirical antibiotic selection process.

## Additional file

Additional file 1: Patient demographics and hospital characteristics. (DOCX 13 kb)

## Abbreviations

CLSI: Clinical and Laboratory Standards Institute
ESBL: Extended-spectrum β-lactamase
MDR: Multidrug resistant
NS: Nonsusceptible
UTI: Urinary tract infection
