# Supplementary material for: Effect of prior receipt of antibiotics on the pathogen distribution and antibiotic resistance profile of key Gram-negative pathogens among patients with hospital-onset urinary tract infections
Source: BMC Infect Dis. 2017 Feb 28;17:176. doi: 10.1186/s12879-017-2270-7 (PMC5329905; doi:10.1186/s12879-017-2270-7)
Supplement: Additional file 1: — Patient demographics and hospital characteristics. (DOCX 13 kb) [file 12879_2017_2270_MOESM1_ESM.docx]

**Additional file 1** Patient demographics and hospital characteristics

| Demographics and characteristics | Overall, *n* (%)  *N* = 5574 |
| --- | --- |
| Age group, years | |
| 0-17 | 35 (0.6) |
| 18-34 | 135 (2.4) |
| 35-54 | 586 (10.5) |
| 55-64 | 827 (14.8) |
| ≥65 | 3991 (71.6) |
| Sex | |
| Female | 3918 (70.3) |
| Male | 1656 (29.7) |
| Race/ethnicity | |
| Black | 903 (16.2) |
| Other | 535 (9.6) |
| White | 4136 (74.2) |
| Primary payer | |
| Charity | 16 (0.3) |
| Commercial—indemnity | 180 (3.2) |
| Direct employer contract | 5 (0.1) |
| Indigent | 18 (0.3) |
| Managed care—capitated | 12 (0.2) |
| Managed care—noncapitated | 460 (8.3) |
| Medicaid—managed care capitated | 17 (0.3) |
| Medicaid—managed care noncapitated | 166 (3.0) |
| Medicaid—traditional | 283 (5.1) |
| Medicare—managed care capitated | 107 (1.9) |
| Medicare—managed care noncapitated | 777 (13.9) |
| Medicare—traditional | 3333 (59.8) |
| Other | 51 (0.9) |
| Other government payers | 45 (0.8) |
| Self-pay | 96 (1.7) |
| Workers’ compensation | 8 (0.1) |
| Admission type | |
| Elective | 1179 (21.2) |
| Emergency | 3046 (54.6) |
| Other | 18 (0.3) |
| Trauma center | 38 (0.7) |
| Urgent | 1293 (23.2) |
| Admission source | |
| Home | 3589 (64.4) |
| Other | 251 (4.5) |
| Skilled nursing facility transfer | 198 (3.6) |
| Transfer | 1536 (27.6) |
| Hospital type | |
| Nonteaching | 2653 (47.6) |
| Teaching | 2921 (52.4) |
| Hospital location | |
| Rural | 364 (6.5) |
| Urban | 5210 (93.5) |
| Geographic region | |
| East North Central | 720 (12.9) |
| East South Central | 289 (5.2) |
| Middle Atlantic | 1037 (18.6) |
| Mountain | 60 (1.1) |
| New England | 326 (5.8) |
| Pacific | 587 (10.5) |
| South Atlantic | 1659 (29.8) |
| West North Central | 448 (8.0) |
| West South Central | 448 (8.0) |
| Hospital size by bed count | |
| 0-100 | 48 (0.9) |
| 100-199 | 424 (7.6) |
| 200-299 | 979 (17.6) |
| 300-499 | 2020 (36.2) |
| ≥500 | 2103 (37.7) |
